# Effectiveness of ultra-rapid (20 min) high-frequency in-situ cardiac arrest simulations in a high-volume operating department – A tool for evaluating and implementing emergency routines

**DOI:** 10.1016/j.resplu.2025.100887

**Published:** 2025-01-31

**Authors:** Anna Sundelin, Anders Stålman, Therese Djärv

**Affiliations:** a Capio Artro Clinic Operation Department Sophiahemmet Stockholm Sweden; b Department of Physiology and Pharmacology, Section of Anaesthesia and Intensive Care, Karolinska Institutet Stockholm Sweden; c Department of Molecular Medicine and Surgery, Karolinska Institutet Stockholm Sweden; d Department of Medicine Solna, Karolinska Institutet Stockholm Sweden; e Emergency Department, Karolinska University Hospital Stockholm Sweden

**Keywords:** Cardiac arrest, Cardiopulmonary resuscitation CPR, Post-resuscitation care, Rapid response systems, Resuscitation simulation, Resuscitation training, In-situ simulation

## Abstract

**Background:**

In-situ simulations are effective in identifying latent safety threats. In high-volume elective operating departments, cardiac arrests are rare due to strict patient selection. Implementing in-situ cardiac arrest simulations in these settings is crucial to evaluate adherence to cardiopulmonary resuscitation (CPR) guidelines and enhance patient safety by detecting and managing safety threats.

**Method:**

From October 2023 to June 2024, a 20-minute in-situ cardiac arrest simulation with debriefing was conducted bi-weekly in a high-volume orthopedic surgery ward with seven operating rooms, without additional staffing. Latent safety threats were identified and addressed. Time to call for help, start of compressions, and first defibrillation was measured, as was airway management choice by anesthesia. Staff confidence was assessed via an anonymous 11-step Likert-scale questionnaire before and after the project, ranging from 0 (no confidence) to 10 (highest confidence).

**Results:**

22 simulations were conducted. Multiple safety improvements were implemented, including role clarification and development of an amiodarone kit. Adherence to cardiopulmonary resuscitation guidelines was strong, achieving time goals in 21 simulations (95%). Anesthesia intubated in 100% of cases when present (16/22, 73%). The questionnaire response rate was 72% (38/53). Staff confidence significantly improved after the project, with median scores increasing from 4.5 to 7.0 (IQR 2.25–7 before, 6–8 after) for personal ability (p < 0.001) and 6.5 to 8.5 (IQR 4–7.25 before, 8–9 after) for team ability (p < 0.001).

**Conclusions:**

A 20-minute in-situ cardiac arrest simulation with debriefing is feasible in a high-volume operating department. Mitigating safety threats and achieving guideline adherence demonstrates functional emergency routines. Staff confidence in managing cardiac arrests significantly increased.

## Highlights

• High-frequency, ultra-rapid in-situ cardiac arrest simulations are feasible in high-volume operating departments without additional staffing.
• Simulations identified and mitigated multiple latent safety threats, including role definition and medication preparation.
• Adherence to CPR guidelines was achieved in 95% of simulations, demonstrating effective emergency routines.
• Advanced airway management with intubation was preferred in 100% of cases where anesthesia personnel were present.
• Staff confidence in managing cardiac arrests significantly improved after simulations (p < 0.001).

## Introduction

In-situ simulations are a proven method for mitigating latent safety threats and are effective educational tools.^1^, ^2^, ^3^, ^4^ At high-volume elective operating departments in local hospitals, the patient population is carefully selected for patient safety reasons, rendering cardiac arrests very uncommon.^5^, ^6^, ^7^, ^8^ It is imperative to implement in-situ cardiopulmonary resuscitation (CPR)-simulations in these settings to evaluate adherence to new CPR guidelines and to increase patient safety, detecting and managing safety threats.^9^, ^10^, ^11^, ^12^, ^13^, ^14^

Timely actions – such as calling for help, initiating CPR, and defibrillation – are critical for increasing survival ratios with favorable neurological outcomes.^15^, ^16^ Guidelines recommend that help be called and CPR initiated within one minute, and defibrillation should occur within three minutes of cardiac arrest.^15^, ^17^, ^18^ Unfortunately, many hospital settings lack clarity on whether these timeframes are consistently met.^19^

Optimal airway management practices during cardiac arrest have been widely debated, particularly in the context of in-hospital cardiac arrest (IHCA), where evidence remains limited.^20^, ^21^, ^22^ According to the 2021 guidelines, endotracheal intubation is recommended as a primary airway management technique for healthcare providers who maintain a minimum 95% success rate within two attempts under typical conditions.^17^ This proficiency is generally achieved after approximately 125 intubations.^23^

Research indicates that confidence enhances the effectiveness of care delivered in emergency situations, where timely and efficient response is critical.^24^ Suboptimal care has been shown to be caused by, for example, a failure of organization, possibly contributing to morbidity and mortality in intensive care admission situations.^25^ In-situ simulations have been shown to aid efficiently in enhancing the structure during emergency situations.^13^, ^26^ In-situ simulations of CPR have been associated with increased confidence and transfer of abilities to manage other emergent situations.^27^, ^28^, ^29^, ^30^, ^31^

The objectives of this study were to implement high-frequency in-situ cardiac arrest simulations to assess and refine protocols for managing cardiac arrests and emergency scenarios. We focused on examining adherence to guidelines concerning airway management decisions by anesthesia teams and response timing. Additionally, the project assessed pre- and post-simulation confidence levels, measuring confidence in individual and team performance of managing cardiac arrest.

## Materials and methods

### The hospital setting

The simulations were conducted at a local hospital without an emergency department, (ED). The hospital has in total 20 operating rooms, (OR), and two wards for elective, post-operative patients with a total of 30 hospital beds. An anesthesiologist is available on call after 6p.m., with an allocated 30-minute response time to arrive at the hospital site. No surgeons are available on site after 5p.m. The operating department in the study consists of seven ORs, and 20 post-operative care beds, open until 7p.m. Patients undergo mainly orthopedic surgery, with half of the ORs for out-of-hospital-care and the other half admitted for elective in-hospital-care post-surgery. After 6p.m. staffing is decreased to one nurse, one nursing assistant and a two-person cleaning and service crew, with no physicians.

### Before the project

Before the project, in September 2023, all personnel were invited to answer an anonymous, voluntary questionnaire – including anesthesiologists, nurse anesthetists, operation nurses, service workers, sterile facility workers, post operative nurses, and nursing assistants from both operation and post operative care. Participants were asked about confidence in managing cardiac arrests – both confidence in their own ability and in the team’s ability, on an 11-step Likert scale ranging from 0 (no confidence) to 10 (highest possible confidence).

All staff were informed about the project and to respond to calls for help and alarms according to the workplace’s routines.

The goal of the project was to implement one simulation every week or every other week, (depending on the availability of instructors and other ongoing projects), during the fall of 2023 and the spring of 2024, with a hiatus for Christmas break.

### Mitigating latent safety threats

Instructors and participants were informed to look for problems and possible solutions, both during the scenarios and afterwards in the debriefing. Expected areas of safety improvement were for example (but not exclusively); the emergency cart and emergency equipment, (do we find what we need to find, is everything in a logical and easily available place, do we have everything we need); roles, communication and documentation, (which roles are adequate in our setting and do they differ throughout the day depending on staffing, is our communication looped and effective, are we documenting correctly and is it manageable); CPR quality, (can we provide good quality CPR in different settings and scenarios, and if not are there any manageable obstacles that can be handled). Instructors documented all observed and mentioned problems and solutions during and immediately after each simulation. Executives and staff responsible for emergency management were informed continuously during the course of the project as safety threats were discovered.

### The simulations

A manikin integrated with the operation ward’s defibrillator (Resusci Anne® QCPR Full Body with Resusci Anne® Airway head and ShockLink^TM^, [Laerdal Medical, Stavanger, Norway]) was prepared according to the scenario, with peripheral venous catheters and monitoring as applicable. SimMon^TM^ medical app ([Castle Andersen ApS, Hillerod, Denmark]) was used to display monitoring. Participants used the real emergency cart with the real documentation sheets. Real, expired adrenaline was used. For other medications SodiumChloride marked “Exercise” was used. Single-use peripheral venous catheters, intraosseous needles, laryngeal masks, tubes, bougies and syringes were used and saved between the simulations. The instructor placed single-use materials on the emergency cart right before each simulation, to be found where the staff normally would find them. These simulations are considered high-fidelity, using a real defibrillator showing live ECG, real-time monitoring changing depending on the interventions, and real airway equipment.

The team of instructors were composed of an anesthesiologist, two assistant nurses, an anesthesia nurse and an intensive care/post-operative nurse. For each simulation 1–4 instructors were present, depending on staffing possibilities and patient care workload.

Simulation started with the instructor preparing the manikin and giving a short instruction to a nurse in charge. The nurse went to his/her patient and the scenario played out. Time was started from when the patient first displayed cardiac arrest symptoms in front of the nurse. All staff were informed about the project but not the exact time or place of the simulations and were expected to answer all calls for help as per usual. Since the preparation and simulation took place somewhere in the operation department, many potential participants knew in advance that there might be an upcoming simulation, (usually within the hour). Timing of call for help, start of CPR and defibrillation was manually registered by the instructor. After each scenario the times were registered in a spreadsheet together with choice of airway management and notes of safety threats or identified improvable ideas. Expected airway management strategies were bag and mask-ventilation, laryngeal mask placement and endotracheal intubation with or without video laryngoscopy. Examples of possible patient scenarios are shown in Table 1.

**Table 1:** Examples of possible patient scenarios.

**Patient**	**Cause of cardiac arrest**
60-year old healthy woman. Before total knee arthroplasty.	Anaphylactic reaction to either antibiotics or NSAID
31-year old elite marathon runner with previous endocarditis. Before anterior cruciate ligament repair.	Primary arrhythmia
70-year old smoker with angina pectoris. After shoulder arthroscopy in Beach position with prolonged hypotension after induction.	Heart attack when going to the bathroom.
55-year old man with atrial fibrillation on anticoagulants. After total hip arthroplasty.	Massive hemorrhage

Executives allocated a maximum of 20 min for each simulation including debriefing, to ensure maintained quality in simultaneous patient care. For simulations the objective was to pursue each scenario to include defibrillations, medication discussions, airway management and a verbalized ABCDE-surey. For debriefing all participants were invited ti share insights. To ensure that the obectives were achieved, instructors chose to divide the time for simulation and debriefing roughly equally.

Scenarios were up to ten minutes with an immediately following hot de-brief of up to ten minutes. De-brief was led by the instructor in a semi-structured way, using the debriefing with good judgement method.^32^ The in-situ simulations were stopped or postponed if there were patient emergencies requiring attention.

Different timeslots were identified for the scenarios, targeting different staff compositions. In the evenings fewer staff are available, and the project sought to investigate airway management, role distribution and adherence to the recommendations for several possible team formations. When simulating evening scenarios all available resources were not allowed to enter the simulation, only the ones corresponding to evening staffing. For all other timeslots all available staff were expected to answer the call for help in case simultaneous patient care was not compromised, between three and 15 individuals from different staff categories.

### After the project

The same questionnaire was offered the same personnel after the project, in June 2024.

### Statistics, data presentation and management

Statistic calculations, graphs and tables were produced using Python version 3.11.3, Python Software Foundation. Normality in the data was evaluated with the Shapiro-Wilk test. P-value was calculated using Mann-Whitney *U* test. Results are presented as frequencies (%), minutes:seconds, or medians with interquartile range (IQR) and range.

### Ethical approval

An advisory opinion (reference number 2023-06315-01) was issued by the Swedish national Ethical Review Authority. They stated that there were no ethical objections towards this research project in that it only gathers anonymous data and is not affecting the research persons. Informed consent was given for each questionnaire. Data is only presented on group level.

## Results

### Implementation of high-frequency in-situ cardiac arrest simulations

In-situ simulations were conducted more than bi-weekly, totaling 22 simulations over 38 weeks. One simulation was prematurely halted due to an emergent situation occurring simultaneously. On five occasions the instructors were engaged in patient care and could not be relieved of their duties. On six occasions an unplanned decrease in staffing due to short-term illnesses made it impossible to conduct the simulations.

### Mitigating latent safety threats

Examples of several latent safety threats and how they were managed are provided in Table 2.

**Table 2:** Examples of several latent safety threats and how they were managed.

**Challenge**	**Before**	**Findings**	**Solution**
Preparing amiodarone	10% glucose and amiodarone were placed with the other medication in the emergency cart	There was difficulty in finding the medication and preparing it correctly in an emergent situation	An amiodarone-glucose-syringe-kit was created and coupled with instructions for preparation
Documentation	Documentation sheet from the Swedish Resuscitation Council	It was difficult to document correctly using the existing documentation sheet	A new documentation sheet customized to our setting, including post-ROSC-documentation support, was developed together with the Swedish Resuscitation Council
Roles	There were no set or decided roles	We found that different roles were needed for different staffing situations	Action cards were developed for different staffing situations roles
Simultaneous emergencies	There was little knowledge among the personnel about how to act when simultaneous emergencies arose	There is a technique to sound an extra strong alarm for situations with simultaneous emergencies	Education about the special alarm
Monitoring	EtCO2-monitoring technique using the defibrillator was not known	EtCO2 was not monitored during or after cardiac arrest	Education on our EtCO2-monitoring possibilities and why it is important
Ergonomics	There was no strategy on how to improve ergonomics to improve chest compressions	Hospital beds were not optimized in height or position to facilitate high quality chest compressions	During the project and action card development this was improved and generally the assistant nursing staff were given this task.
Aortic compression	To apply external aortic compression for massive lower body hemorrhage	There was no existing routine	During the project and action card development this was improved. The action was generally decided by the anesthesiologist, and the task given to one of the available personnel.
Teamwork	As emergency situations are relatively rare, the need for big teams from all occupational groups are small	The different occupational groups were not sure how they could best cooperate and contribute to the cardiac arrest situations.	During the project the strengths and possibilities within and between the occupational groups became more evident and the teamwork improved, both as seen from an instructor point of view and mentioned in the debriefings.
Defibrillation safety	To defibrillate with minimal break in chest compressions at the same time as ensuring the personnel’s safety	With many people close to the patient it was difficult to minimize the break to perform safe defibrillations	All personnel were educated to back away (if applicable) and show palms up that they were not in risk of being defibrillated. The shortening of the break in compressions was difficult to measure, but it was perceived as a safer routine for all staff including the one responsible for defibrillating.

### Adherence to guidelines

**Fig. 1:**
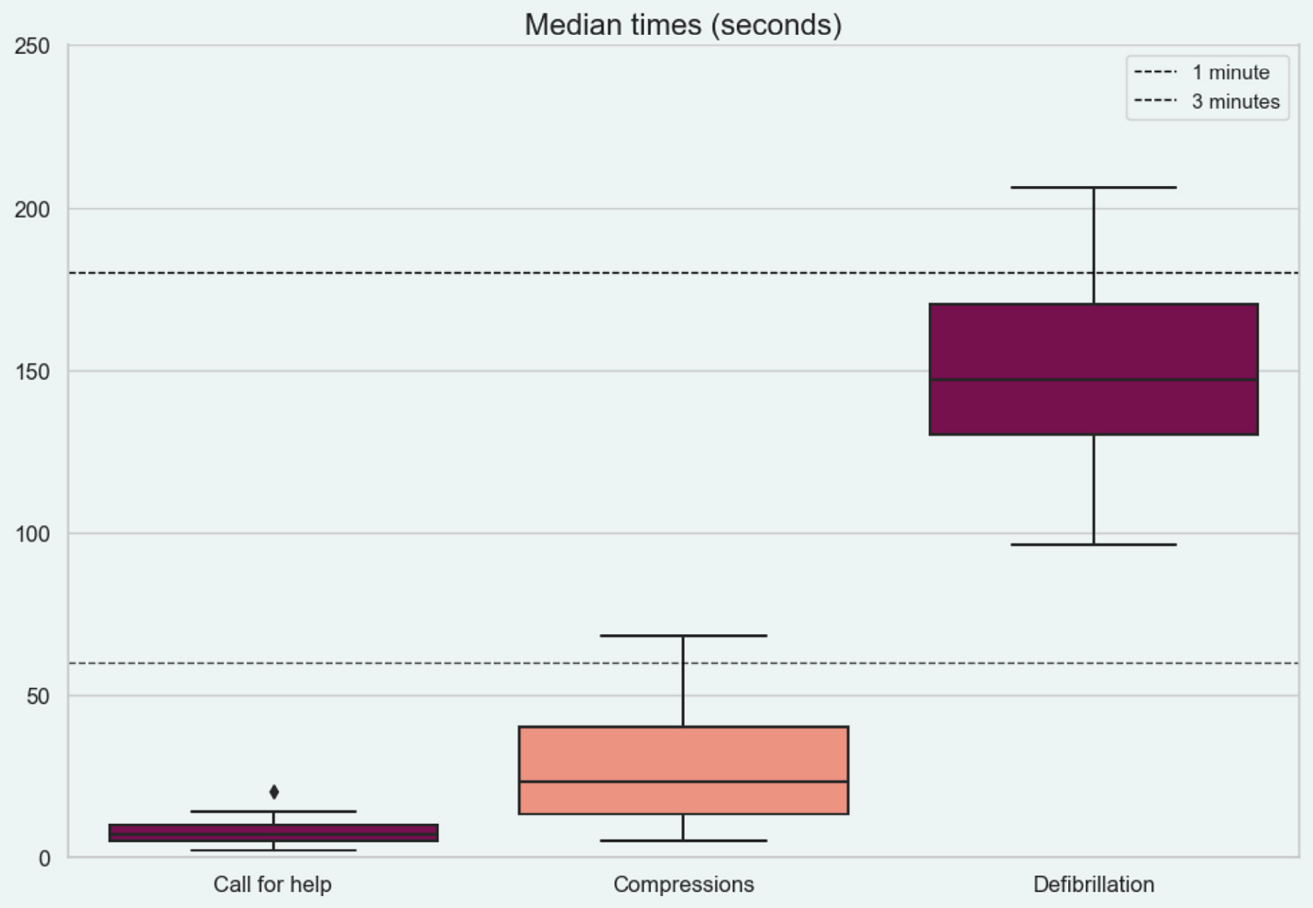
**Response times.** Boxplot showing time from cardiac arrest to call for help, start of CPR and defibrillation. Recommendations state 1 min and 3 min respectively, as shown on dotted lines across.

Time to call for help, start of CPR and defibrillation was within recommended guidelines for all cases except one, (Fig. 1, Appendix table A1). In that simulation the manikin was found on the floor of the bathroom, behind the toilet, which prolonged the time to intervention. Even in this special case the goals of response and treatment times were almost reached, with an eight second delay to CPR, and a 26 s delay to the first defibrillation. In some scenarios the patients were ECG-monitored to mimic a true perioperative setting, which could explain time to call for help and start of compressions being very short.

### Airway management

Airway management was 16 cases of intubation (73%) which corresponded to 100% of the times where intubation skill was available. No one attempted a laryngeal mask. In six cases (27%) bag-mask was used to ventilate. Bag-mask or mouth to mouth and then bag-mask was the first choice in all scenarios. Video laryngoscope was used in 50% of cases. Time of intubation and secured airway varied between 2.5 and 7 min from cardiac arrest. Intubation was generally initiated after the emergency cart arrived and after the rhythm was analyzed, (and if applicable defibrillated).

### Confidence

**Fig. 2:**
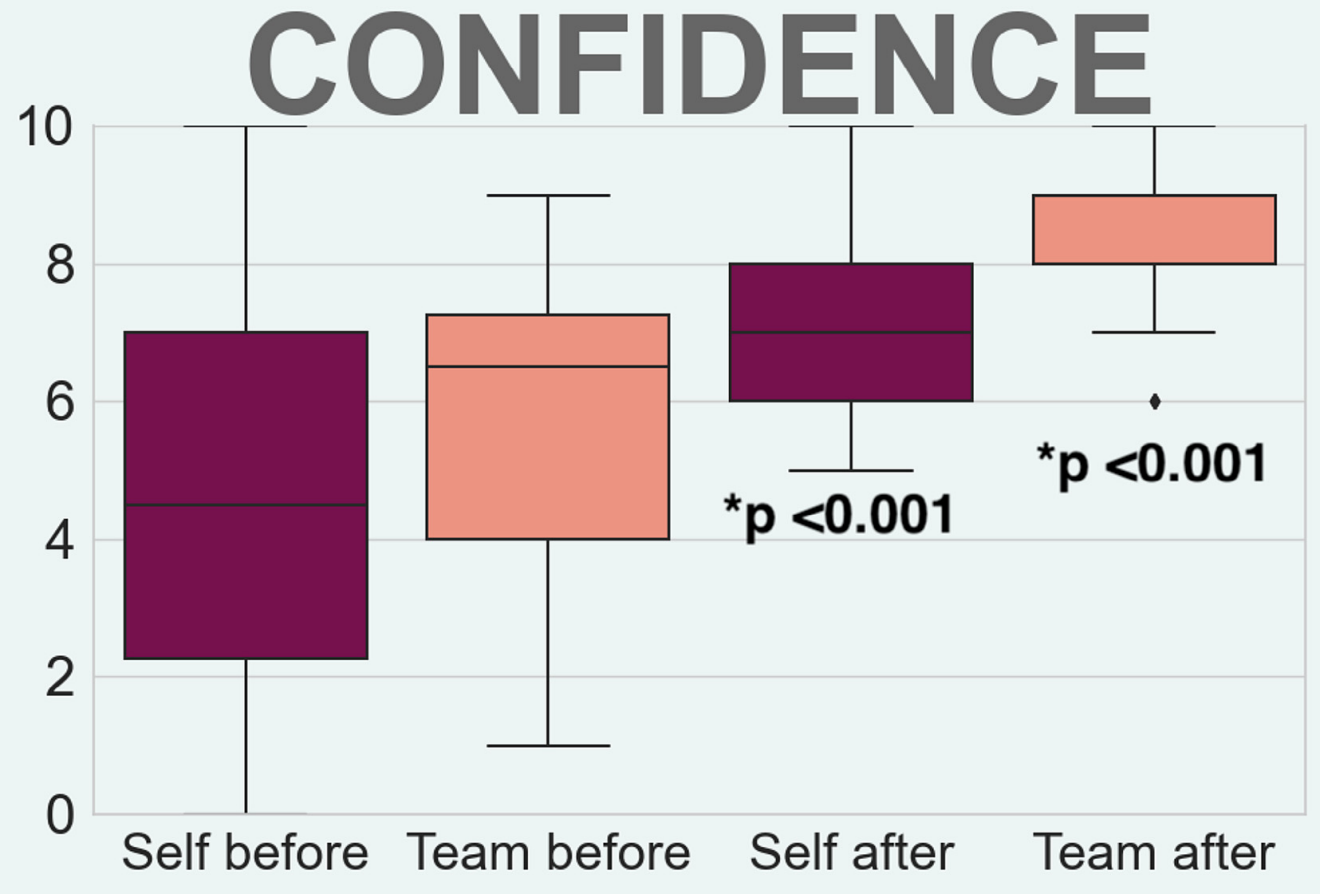
**Confidence levels.** There was a statistically significant increase in confidence before and after the project, both in the participants own ability in managing cardiac arrest, as well as the confidence in the team’s ability. The confidence in the team’s ability is higher, with a median of 9 (IQR 8–9) after the project.

Response rate of the questionnaire was 72% (38/53). Confidence was improved (p < 0.001*) between the start and the end of the project, (Fig. 2). For details on median, IQR and range see Appendix Table A2.

## Discussion

### Summary of key findings

The project demonstrated the feasibility and effectiveness of implementing regular in-situ cardiac arrest simulations in a high-volume operating facility. The simulations assisted in mitigating several latent safety threats. Our findings show that our routines are effective, allowing us to provide high quality CPR during all operating hours with different team compositions, including advanced airway management when anesthesia personnel are present. Additionally, the in-situ simulations have significantly improved the confidence of the personnel in managing cardiac arrests.

### Comparison with existing literature

Our findings are consistent with prior studies that demonstrate the value of in-situ simulations in enhancing emergency preparedness and mitigating latent safety threats.^4^ For example, research by Lighthall et al highlighted the effectiveness of regular simulation training in improving adherence to resuscitation guidelines, which aligns with our 95% adherence rate during simulations.^10^

A novel aspect of our study is the integration of high-frequency simulations in a high-volume elective surgical setting without an emergency department, an environment often overlooked in emergency preparedness research.

Additionally, our identification and resolution of specific latent safety threats, such as the development of an amiodarone kit and improved documentation templates, add practical value to existing literature.

Interestingly, there was no observable reduction in response times over the course of the project, possibly due to the increased complexity of scenarios introduced later in the study. This aligns with findings in a 2025 ILCOR review, which emphasizes the importance of scenario complexity on training outcomes.^4^

### Mitigating latent safety threats

The National Swedish Resuscitation Council offers a template for documenting during CPR. After the publishing of the latest guidelines in 2021 there is no specific template suggested for post-ROSC-documentation. Gradually post-ROSC-care was improved and given more focus throughout the project.

### Confidence

The confidence in the team’s ability to manage cardiac arrest is bigger than the confidence in the individual ability. This is supported in previous literature, suggesting that the collective knowledge and skills of a team are greater than those of an individual.^27^

### Limitations

The study has some limitations, including medium–high response rate and potential bias in the evaluation of airway management due to the manikin’s facial appearance, which may be easier to intubate. The increase in confidence could be due to more confident personnel responding to the second survey.

### Future research directions

An important and relevant future direction of research would be to investigate the influence of simulation on more relevant outcomes, such as knowledge retention, clinical practice, and patient outcomes. Future studies should also explore the impact of different simulation frequencies and durations on emergency preparedness and staff confidence. A 2025 ILCOR review emphasizes the need for research on resources and methods for implementing and maintaining in-situ simulation programs for resuscitation training.^4^

## Conclusions

It is feasible to implement ultra-rapid, high-frequency in-situ cardiac arrest simulations in a high-volume operating department. Adherence to guidelines, emergency routines and airway management are evaluated through in-situ simulations. The confidence levels for managing cardiac arrest increase significantly.

## Lessons learned

• Implementing a 20-minute in-situ simulation once every other week is feasible and effective.
• Using simulations to evaluate and improve routines for standard time-critical scenarios is valuable.
• Intubation is the chosen airway management technique by anesthesia.

## Availability of data and materials

All data used and analyzed in this study is available from the first author upon reasonable request.

## Author contributions

Idea generation by ASu, supported by TD. ASu drafted the ethical application, questionnaire, and manuscript, gathered, and made the first analyses and statistical calculations of the data. TD and ASt revised the ethical application, questionnaire, and manuscript. ASu is the corresponding author.

## CRediT authorship contribution statement

**Anna Sundelin:** Writing – review & editing, Writing – original draft, Project administration, Methodology, Investigation, Funding acquisition, Formal analysis, Data curation, Conceptualization. **Anders Stålman:** Writing – review & editing, Supervision. **Therese Djärv:** Writing – review & editing, Supervision, Methodology, Conceptualization.

## Funding

ASu received a grant (6000€) from the Swedish Resuscitation Council to buy the manikin, airway head, ShockLink^TM^ and monitoring screen.

## Declaration of competing interest

The authors declare that they have no known competing financial interests or personal relationships that could have appeared to influence the work reported in this paper.
