# Supplementary material for: Effectiveness of ultra-rapid (20 min) high-frequency in-situ cardiac arrest simulations in a high-volume operating department – A tool for evaluating and implementing emergency routines
Source: Resusc Plus. 2025 Jan 31;22:100887. doi: 10.1016/j.resplu.2025.100887 (PMC11847464; doi:10.1016/j.resplu.2025.100887)
Supplement: Supplementary Data 1 [file mmc1.docx]

# Appendix

# Effectiveness of Ultra-Rapid (20 minute) High-Frequency In-Situ CPR-Simulations in a High-Volume Operating Department – a Tool for Evaluating and Implementing Emergency Routines

Anna Sundelin^1,2^, Anders Stålman^1,3^, Therese Djärv^4,5^

1. Capio Artro Clinic Operation Department, Sophiahemmet, Stockholm, Sweden.
2. Department of Physiology and Pharmacology, Section of Anaesthesia and Intensive Care, Karolinska Institutet, Stockholm, Sweden.
3. Karolinska Institutet, Stockholm, Sweden.
4. Department of Medicine Solna, Karolinska Institutet, Stockholm, Sweden.
5. Emergency Department, Karolinska University Hospital, Stockholm, Sweden.

**Table A1**. Time from cardiac arrest to call for help, start of CPR and defibrillation, (minutes:seconds).

| **(Minutes:seconds)** | **Median** | **IQR** | **Range** |
| --- | --- | --- | --- |
| **Call for help** | 0:07 | 0:05-0:10 | 0:02-0:20 |
| **Start of CPR** | 0:23 | 0:13-0:40 | 0:05-1:08 |
| **Defibrillation** | 2:27 | 2:10-2:50 | 1:36-3:26 |

**Table A2**. Confidence was anonymously reported on an 11-step Likert scale ranging 0-10 before and after the project, response rate 72%. Participants were asked to rate their confidence in their own ability and the team’s ability to manage cardiac arrest. Results are presented as median, IQR and range. A p-value of <0.05 was considered statistically significant. *Statistically significant increase in confidence after the project.

|  | **Before** (median;(IQR);[range]) | **After** (median;(IQR);[range]) | **p-value** |
| --- | --- | --- | --- |
| **Own ability** | 5.5 ; (2.25-7) ; [0-10] | 7 ; (6-8) ; [5-10] | <0.001* |
| **Team’s ability** | 6.5 ; (4-7.25) ; [1-9] | 9 ; (8-9) ; [7-10] | <0.001* |

### Expanded description of mitigated latent safety threats.

#### Latent safety threats

During the project several issues were detected and handled.

##### Medication

A separate packaging for amiodarone together with syringes, glucose and an instruction was prepared and placed in the emergency cart.

##### Documentation

A sheet for documentation was developed and iterated to suit our setting. The documentation sheet contains two sides, one for cardiac arrest and one for return of spontaneous circulation (ROSC). In the latest guidelines management after ROSC was given more focus^27^. The simplest approvement to our emergency cart was attaching a pen to the clipboard with the emergency documentation sheets, making the process of documenting easier.

##### Roles

In the beginning of the project, the nurse in charge often began compressions and stayed there for several minutes. This made giving a recap of the patient and what had happened very difficult. The team practiced taking over compressions early and releasing the first on scene to give report.

Action cards were developed, for different scenarios and staffing. Daytime there are several anesthesiologists and anesthesia nurses available for alarm calls. In the evenings there are sometimes only one post-operative nurse, a nursing assistant and two staff members from service. This makes the possibilities for roles differ during the shifts. Roles were iterated throughout the project, considering difference in staffing.

##### Simultaneous emergencies

On one occasion there was a real emergency at the same time as the simulation. Our alarm buttons have a function to send an extra strong and loud alarm signal in those cases. This knowledge was new to most of the staff, and now everybody knows how to activate it and how it sounds.

##### Monitoring

Monitoring of end-tidal carbon dioxide (E_t_CO_2_) has a stronger recommendation in the new (2021) guidelines^15^. During the project we saw that in the beginning E_t_CO_2_was almost never discussed. At the later stages of the project E_t_CO_2_-monitoring was activated early in almost every cardiac arrest simulation. It was also reflected upon during CPR and after ROSC.

##### ECG and aortic compression

The prevalence of early 12-lead electrocardiogram (ECG) after ROSC also increased during the project, as well as aortic compressions for simulations with suspected massive hemorrhage.

##### Ergonomics

To perform high quality CPR and chest compressions, ergonomics are important ^28^. During the project we saw an increase in the team helping each other achieve optimal conditions for CPR – lowering and flattening the bed, removing pillows, bringing a stool, moving the bed out from the wall to make room for airway management.

##### Team spirit

A common comment in the hot de-brief was the increasing team spirit in the workplace – especially between professional groups that don’t usually work together.

##### Safety

The defibrillations were increasingly safe for the staff and the pause in compressions shortened, due to defibrillations being practiced regularly and every participant showing the palm of their hands during the defibrillator’s charging and defibrillation period.
